# Adding Ezetimibe to High-Intensity Statin at the Time of Admission in Patients With Acute Coronary Syndrome: Results From the Optimization of Low-Density Lipoprotein Cholesterol After Acute Coronary Syndrome (OLA) Study

**DOI:** 10.7759/cureus.89513

**Published:** 2025-08-06

**Authors:** Kunal Mahajan, Aditya Batra, Ashu Gupta, Saurabh Arora, Jai Bharat Sharma, Surender Himral, Deep Dutta

**Affiliations:** 1 Cardiology, Himachal Heart Institute, Mandi, IND; 2 Cardiology, Holy Heart Advanced Cardiac Care and Research Centre, Rohtak, IND; 3 Endocrinology, Diabetes, and Metabolism, Cedar Superspeciality Healthcare, New Delhi, IND

**Keywords:** acute coronary syndrome, combination therapy, ezetimibe, ldl-c, statin

## Abstract

Background: Achieving rapid and substantial reductions in low-density lipoprotein cholesterol (LDL-C) soon after acute coronary syndrome (ACS) is associated with improved cardiovascular outcomes. Current guidelines recommend high-intensity statin therapy, yet many patients fail to reach LDL-C targets early, putting them at continued risk. The present study investigates whether upfront combination therapy with rosuvastatin 40 mg and ezetimibe 10 mg, started at admission, is more effective and efficient in achieving early LDL-C goals compared to statin monotherapy in statin-naïve ACS patients.

Materials and methods: In this single-center prospective study, statin-naïve patients presenting with ACS and undergoing percutaneous coronary intervention were started on combination therapy (rosuvastatin 40 mg plus ezetimibe 10 mg, n=63). Lipids were measured at baseline and one, two, four, and six weeks. Results were compared to a retrospective, matched cohort (n=61) treated with rosuvastatin 40 mg monotherapy, who had complete baseline and week 4 lipid profiles. The primary endpoint was the percent LDL-C reduction at four weeks post-ACS and the proportion of patients achieving LDL-C targets of <50 mg/dl and <70 mg/dl by four weeks. Secondary endpoints included kinetics of LDL-C lowering, safety, and tolerability.

Results: Baseline LDL-C was similar between groups (115.2 ± 29.3 vs. 118.8 ± 35.4 mg/dl, p=0.65). At four weeks, mean LDL-C was 43.4 ± 12.2 mg/dl (combination) and 65.3 ± 25.3 mg/dl (monotherapy), signifying reductions of 62.3% and 45.1%, respectively (p=0.04). LDL-C targets of <50 mg/dl and <70 mg/dl were achieved at substantially higher rates in the combination group (74.6% vs. 27.9% and 95.2% vs. 59.1%, respectively; both p<0.0001). Maximal LDL-C reduction occurred within two weeks and persisted to six weeks. Combination therapy was safe and well-tolerated, with only mild myalgias observed in 19% of patients, none requiring treatment cessation.

Conclusions: Upfront initiation of rosuvastatin plus ezetimibe in ACS achieves rapid, profound, and sustained LDL-C lowering, with significantly higher rates of target attainment than high-intensity statin monotherapy. Early combination therapy represents an accessible, pragmatic, and cost-effective strategy for high-risk patients, especially in resource-limited environments.

## Introduction

The acute phase following an episode of acute coronary syndrome (ACS), comprising unstable angina, non-ST-elevation myocardial infarction (NSTEMI), and ST-elevation myocardial infarction (STEMI), represents a period of wildly increased vulnerability to recurrent ischemic events, arrhythmias, and heart failure [1]. The biology of this heightened risk is multifactorial: persistent atherosclerotic inflammation, plaque instability, systemic hypercoagulability, and elevated atherogenic lipoproteins all converge to create a scenario in which, if left unchecked, the likelihood of suffering another major event is several-fold greater than at any other period in a patient’s clinical course [1-3].

Guideline-directed management of ACS now universally emphasizes rapid and aggressive modification of risk factors, with cholesterol control considered a central pillar of secondary prevention [4]. The benefits of early, profound low-density lipoprotein cholesterol (LDL-C) lowering have been robustly demonstrated; each mmol/L decline in LDL-C yields approximately a 22% reduction in major adverse cardiovascular events, and these benefits accrue rapidly, especially in the weeks immediately following ACS [1,4]. Not only do high-intensity statins (HIS) remain first-line therapy, but there is increasing appreciation that for many patients, especially those with high baseline LDL-C, diabetes, polyvascular disease, or familial hypercholesterolemia, even maximally tolerated statin doses may prove insufficient for achieving early guideline-mandated LDL-C targets [5].

The Lipid Association of India (LAI), keeping in mind the ethnically heightened risk and characteristic lipid profiles of South Asian patients, has issued a bold recommendation: for all ACS patients, LDL-C must be reduced to below 50 mg/dl as rapidly as possible [5]. To facilitate these ambitious goals, LAI proposes the up-front addition of ezetimibe, a cholesterol absorption inhibitor, to HIS upon hospital admission, not waiting for subsequent assessments of statin effectiveness. This position is supported by accumulating evidence that “the earlier the better,” as maximal benefit is likely to be realized only when LDL-C targets are achieved during the first post-ACS weeks and not at a later time when the risk has already peaked [5].

While international guidelines, such as the European Society of Cardiology and others, have gradually moved in a similar direction, most clinical trials and real-world registries still reflect a paradigmatic inertia; non-statin agents are added only after an initial trial of HIS alone, often weeks after presentation [1,4]. In landmark studies such as IMPROVE-IT, ezetimibe was typically initiated several days or weeks after ACS [6], leaving open the key clinical question: what is the effect of truly simultaneous statin-ezetimibe therapy started from the outset? Cost and access represent additional considerations, particularly for patients in low- and middle-income settings. Whereas PCSK9 inhibitors and novel agents offer additional LDL-C reduction, their prohibitive cost and parenteral administration are barriers to broad implementation. By contrast, ezetimibe is widely accessible, affordable, and orally administered, with decades of safety data [6,7].

We therefore hypothesized that statin-naïve patients treated from the time of ACS admission with a combination of rosuvastatin 40 mg and ezetimibe 10 mg daily would achieve greater and more rapid LDL-C reductions and higher rates of LDL-C target attainment at four weeks than an otherwise comparable cohort who received high-intensity statin alone. We further sought to characterize the safety and tolerability of this approach to inform practical implementation in routine clinical care.

## Materials and methods

Study design and setting

Optimization of LDL-C after ACS (OLA) was a real-world, single-center, prospective observational study with a retrospective control, performed at a tertiary cardiac care center in northern India. The study protocol was reviewed and approved by the Institutional Ethics Committee of the Holy Heart Advanced Cardiac Care and Research Institute (approval number: HH23/2023) and conducted following the principles of the Declaration of Helsinki [8].

Patient selection

All consecutive adult patients (up to 85 years) presenting with a diagnosis of ACS: NSTEMI, STEMI, or unstable angina, between January and March 2023, who underwent PCI, were considered for enrollment. The inclusion criteria for the study were as follows: patients had to be statin-naïve, meaning they had not received any statin or non-statin lipid-lowering therapy in the preceding six months; they must have undergone successful PCI for ACS; they were required to be able and willing to provide written informed consent; and they needed to agree to periodic outpatient follow-up visits and blood sampling for scheduled lipid analysis. Exclusion criteria included prior statin or other lipid-lowering drug use within six months, contraindications to statin or ezetimibe therapy, severe hepatic (hepatic enzymes aspartate/alanine transaminases >3× upper limit) or renal dysfunction (eGFR < 30 ml/min/1.73 m²), need for staged PCI, hemodynamic instability, or anticipated inability to comply with the follow-up protocol.

Treatment protocol (combination arm)

Enrolled patients (n=68) in the study received a combination of rosuvastatin 40 mg daily and ezetimibe 10 mg daily, with therapy initiated immediately upon admission for ACS. In addition to this lipid-lowering regimen, all patients were provided with standard-of-care post-PCI medications, including dual antiplatelet therapy, beta-blockers, and renin-angiotensin system inhibitors as recommended by current guidelines and tailored to individual clinical circumstances. Furthermore, participants received lifestyle and dietary advice to support cardiovascular health and optimize treatment outcomes.

Blood was drawn for a fasting lipid profile at admission (baseline) and one, two, three, four, and six weeks after discharge. At each follow-up, patients underwent a structured symptom review, physical exam, and medication compliance check (pill count, patient self-report). Patients who either withdrew consent or were lost to follow-up before week 4 (n=5) were excluded from the final efficacy analysis; 63 patients completed the protocol and were included as Group 1.

Retrospective comparator cohort (statin arm)

As a comparator, the study used a retrospectively identified cohort of statin-naïve ACS patients (n=61) frequency-matched for age, sex, and diagnostic category, who had presented between October and December 2022, undergone PCI, and received standard post-ACS care including rosuvastatin 40 mg daily without ezetimibe. These patients had baseline and four-week fasting lipid values available in the hospital electronic database; none had non-statin LDL-lowering therapy during follow-up. Follow-up and event tracking were performed per center protocol.

Laboratory methods

Laboratory methods in the study involved direct measurement of LDL-C using standard enzymatic and colorimetric techniques performed in an accredited laboratory. In cases where patients reported symptoms such as myalgias, fatigue, or jaundice, assessments of creatine kinase (CK) and liver function were conducted to monitor for potential adverse effects. Importantly, laboratory personnel were blinded to the treatment groups to minimize bias and ensure the integrity of the laboratory assessments.

Outcome measures

The primary endpoints of the study were the percentage change in LDL-C, defined as ((LDL-C at baseline - LDL-C at 4 weeks)/LDL-C at baseline) × 100, from baseline to week 4, and the proportion of patients achieving LDL-C levels below 50 mg/dl and 70 mg/dl at week 4. Secondary endpoints included the proportion of patients reaching these LDL-C targets at weeks 1, 2, 3, and 6; the detailed time course of LDL-C reduction and target attainment; and safety outcomes such as the incidence, severity, and management of adverse effects, including myalgias (patient-reported muscle pain), hepatic dysfunction (enzyme elevation >3× upper limit), CK elevation (enzyme elevation >3× upper limit), and treatment discontinuation rates.

Statistical analysis

Continuous variables were reported as mean ± standard deviation; categorical variables as frequencies/percentages. Between-group comparisons for continuous variables used Student’s t-tests; categorical variables were compared with a chi-square test. Changes in LDL-C from baseline over time were analyzed using repeated measures ANOVA. All analyses were performed in SPSS Statistics version 27 (IBM Corp. Released 2020. IBM SPSS Statistics for Windows, Version 27.0. Armonk, NY: IBM Corp.) and cross-checked independently by two statisticians. A two-sided p-value < 0.05 was considered significant.

## Results

Baseline demographics and clinical characteristics

The two groups were well-matched for age, sex, prevalence of diabetes, hypertension, and smoking history, as well as clinical presentation (NSTEMI, STEMI, unstable angina) (Table 1). The mean age in the combination group was 59.2 ± 11.1 years; 77.8% were male, 30.2% had diabetes, 39.9% had hypertension, and 41% were current smokers.

**Table 1:** Comparison of baseline characteristics and lipid outcomes of group 1 (ezetimibe + posuvastatin) vs. group 2 (rosuvastatin only) * t = t-statistic (two-sample t-test, used for continuous variables); χ² = chi-square statistic (used for categorical variables) # continuous variables have been expressed as mean ± SD BMI: body mass index, FU: follow-up, LDL-C: low-density lipoprotein cholesterol, SD: standard deviation, STEMI: ST-elevation myocardial infarction

Characteristic	Group 1 (n=63)	Group 2 (n=61)	p-value	Test statistic*
Age in years^#^	54.6 ± 10.9	54.2 ± 10.1	0.60	t = 0.21
Male gender	49 (77.8%)	55 (90.2%)	0.02	χ² = 3.53
Female gender	14 (22.2%)	6 (9.8%)		
Diagnosis STEMI	37 (58.7%)	28 (45.9%)	0.20	χ² = 2.03
Diabetes mellitus	19 (30.2%)	11 (18.1%)	0.38	χ² = 2.49
Hypertension	25 (39.9%)	18 (29.5%)	0.08	χ² = 1.40
BMI >25 Kg/m^2^	33 (52.4%)	38 (62.3%)	0.20	χ² = 1.28
LDL-C at admission, in mg/dl^#^	115.2 ± 29.3	118.8 ± 35.4	0.65	t = -0.62
LDL-C at 4 weeks, in mg/dl^#^	43.4 ± 12.2	65.3 ± 25.3	<0.0001	t = -6.11
% reduction in LDL-C^#^	62.3 ± 12.1%	45.1 ± 11.6%	0.04	t = 8.08
LDL-C <50 mg/dl at 4 weeks FU	47 (74.6%)	17 (27.9%)	<0.0001	χ² = 27.2
LDL-C <70 mg/dl at 4 weeks FU	60 (95.2%)	36 (59.1%)	<0.0001	χ² = 23.3

Baseline LDL-C was similar: mean LDL-C was 115.2 ± 29.3 mg/dl in the combination group and 118.8 ± 35.4 mg/dl in the statin-only group (p=0.65). Other lipid parameters (HDL-C, non-HDL-C, triglycerides) did not differ significantly between groups. All patients underwent successful PCI with drug-eluting stent placement, received standard medical therapy post-ACS, and completed at least one month of event-free follow-up.

Lipid lowering and target attainment

**Figure 1:**
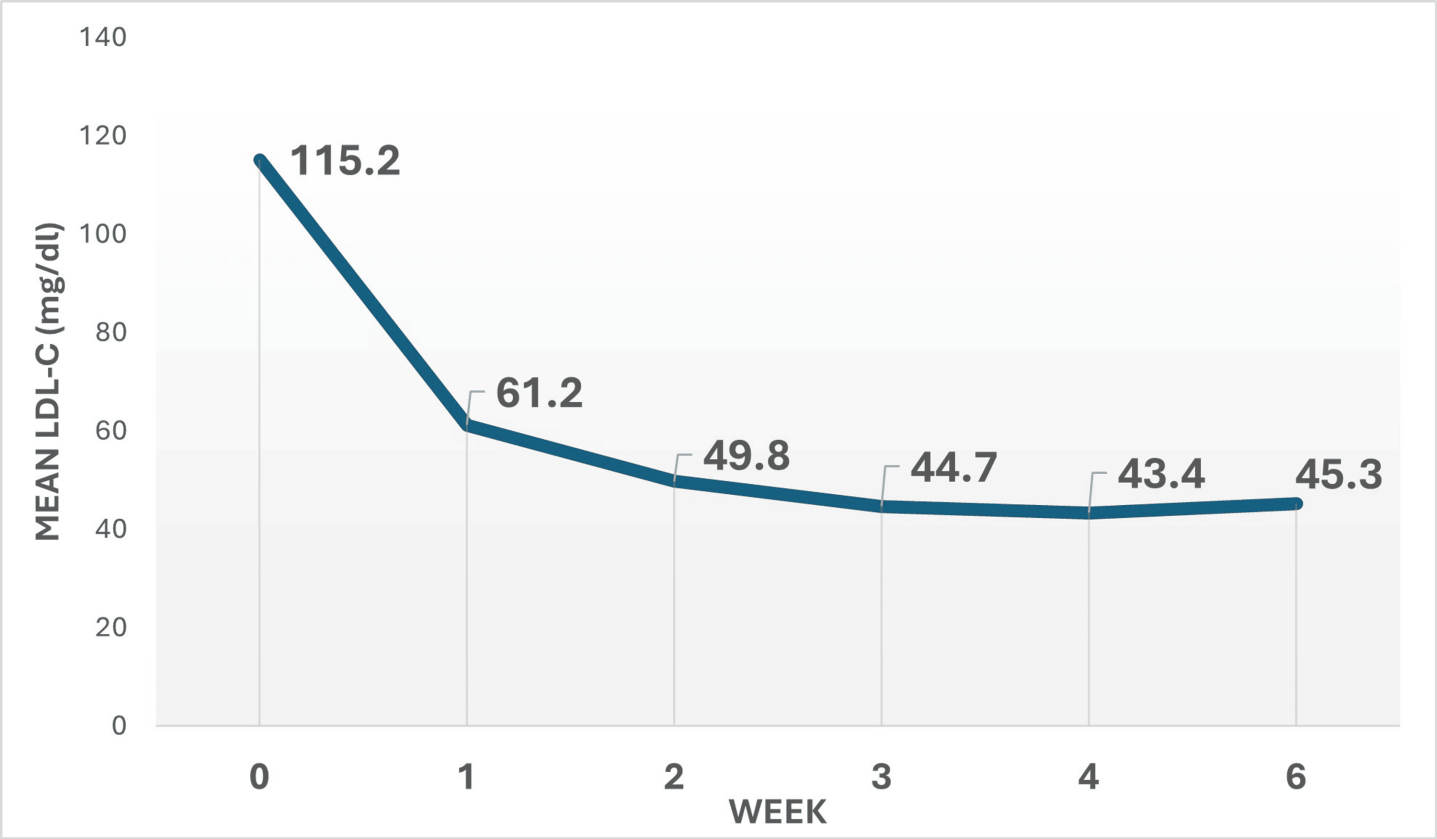
Weekly change in the mean LDL-C LDL-C: low-density lipoprotein cholesterol

**Figure 2:**
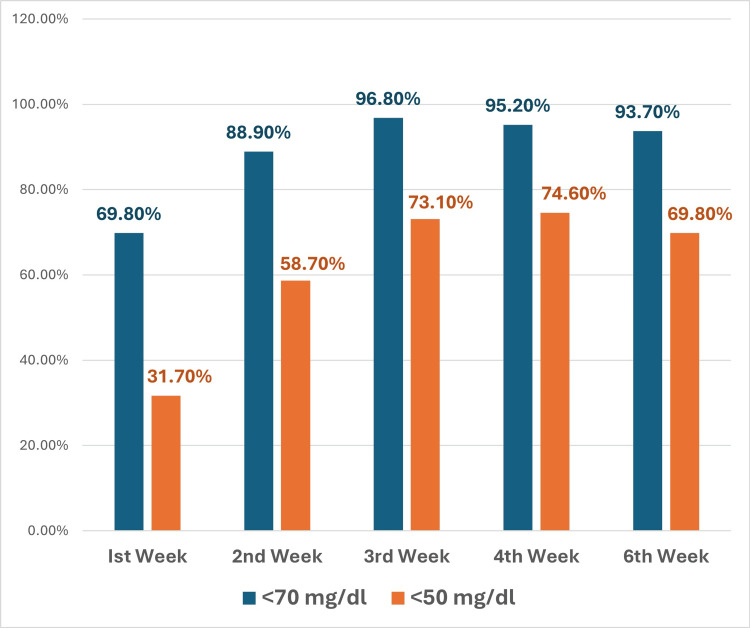
Bar diagram showing week-wise proportion of patients achieving target LDL-C LDL-C: low-density lipoprotein cholesterol

**Figure 3:**
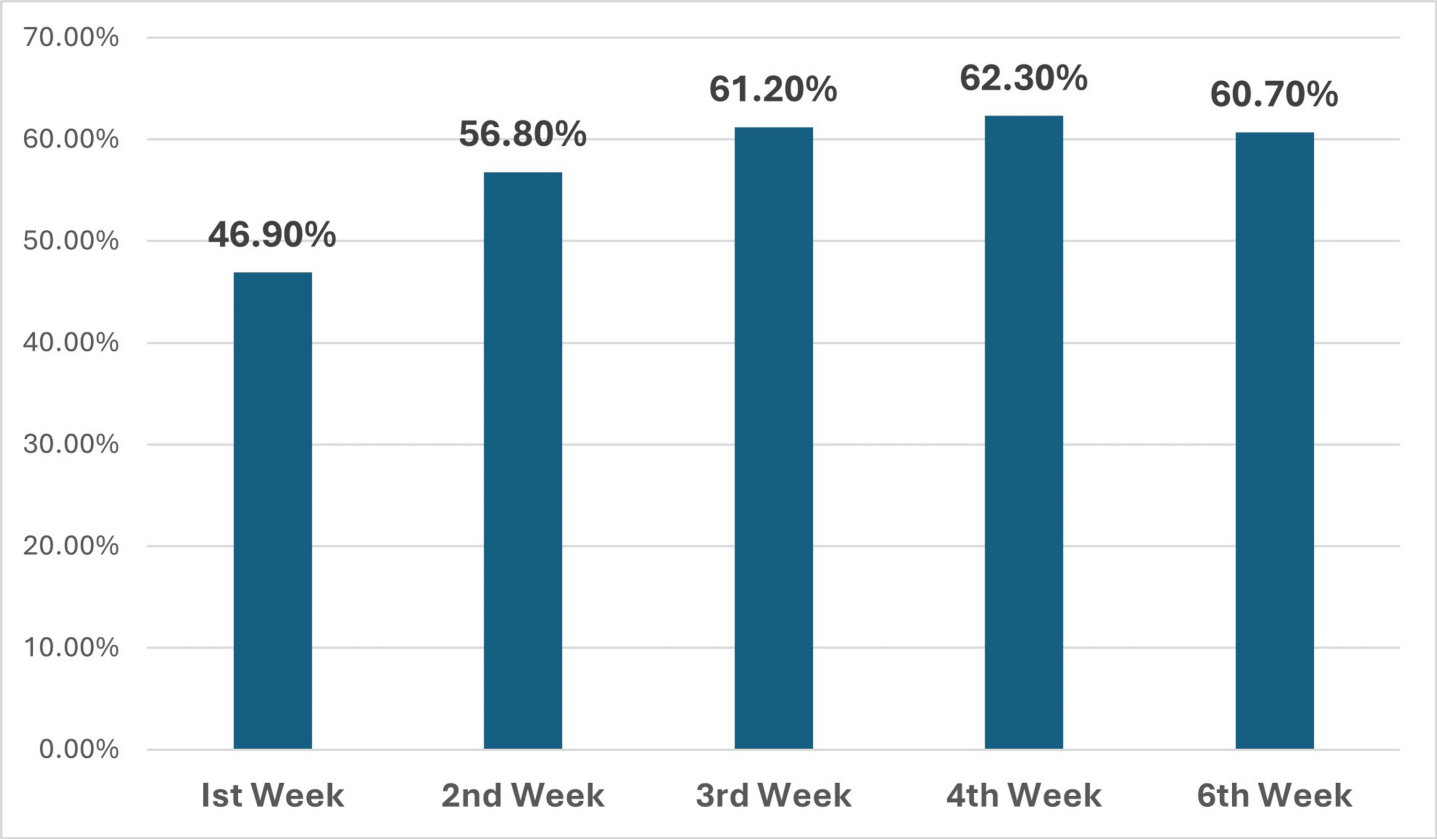
Week-wise percentage reduction in the mean LDL-C compared to baseline value LDL-C: low-density lipoprotein cholesterol

Combination lipid-lowering therapy with rosuvastatin plus ezetimibe demonstrated a significantly greater and more rapid reduction in LDL-C compared to statin-only treatment. At four weeks, the combination group achieved a mean LDL-C reduction of 62.3 + 12.1% from baseline (to 43.4 ± 12.2 mg/dl), while the statin-only group attained a 45.1 + 11.6% reduction (to 65.3 ± 25.3 mg/dl), amounting to an absolute difference of 21.9 mg/dl in favor of the combination (p=0.04). Notably, maximal LDL-C lowering was observed as early as two weeks and was sustained through six weeks in the combination group (Figure 1). Target LDL-C attainment rates were markedly higher in the combination group as well, with 74.6% achieving LDL-C <50 mg/dl (vs. 27.9% with statin-only, p<0.0001) and 95.2% reaching LDL-C <70 mg/dl (vs. 59.1%, p<0.0001) at four weeks (Figure 2). By two weeks, the combination group outperformed statin (58.7%), and 88.9% of patients had achieved LDL-C <50 mg/dl and LDL-C <70 mg/dl, respectively (Figure 2). The kinetics of LDL lowering showed that about 90% of the maximal response was achieved by two weeks for most patients (Figures 1-3), indicating the importance of early follow-up and monitoring. Regarding safety, 19.0% of patients in the combination group reported mild, self-limited myalgias, and 12.7% developed asymptomatic elevations in CK, but no patients discontinued therapy. There were no significant elevations in liver enzymes or creatinine, and no cases of rhabdomyolysis, hepatic failure, or serious adverse events such as ischemic events, arrhythmias, or sudden cardiac death over the six-week follow-up. Overall, combination therapy with rosuvastatin and ezetimibe provided more robust and rapid LDL-C lowering and target attainment than statin monotherapy, with a favorable short-term safety profile.

## Discussion

The primary findings of the OLA study demonstrated greater and more rapid LDL-C reduction with the upfront statin-ezetimibe combination compared to statin monotherapy. The reductions were statistically robust, with significant differences in both mean percentage and absolute reductions at four weeks, and a higher proportion of patients achieving LDL-C targets. These results were consistent across pre-specified subgroups (Table 1), increasing confidence in the directionality and magnitude of the observed effect. The short timeframe and repeated LDL-C measurement post-ACS support a temporal association between intervention and lipid response. However, the study’s observational design precludes definitive causal conclusions. While we matched groups on key known confounders and used standardized follow-up, unmeasured confounding cannot be excluded. Inferences regarding clinical events (e.g., myocardial infarction, mortality) are based on prior evidence linking LDL-C reduction to improved outcomes, not directly observed endpoints in this study.

Nevertheless, the present study provides strong real-world evidence supporting the early, upfront combination of high-intensity rosuvastatin and ezetimibe in ACS. Compared to the standard approach of initiating high-intensity statin alone, this strategy led to more than 15% greater mean LDL-C reduction, with nearly three-quarters of patients achieving the aggressive LAI target of <50 mg/dl and >95% reaching the ACC goal of <70 mg/dl at just four weeks.

Early reduction in LDL-C is not simply a laboratory metric but has tangible clinical benefits [1-3]. Multiple meta-analyses and clinical trials have shown that aggressive LDL-C lowering in the days to weeks after ACS translates into lower rates of reinfarction, urgent revascularization, stroke, and cardiovascular mortality [1,7]. Newer evidence suggests the risk curve for recurrence is particularly steep in the first month, so delays in therapy or goal attainment may squander the period of most significant potential benefit [2,3].

While the addition of non-statin agents such as PCSK9 inhibitors can achieve greater LDL-C lowering, their cost, injectable nature, and limited availability restrict routine use, particularly in resource-constrained settings [9]. Ezetimibe is a logical, cost-effective, and widely available choice, supported by outcome studies and recent guideline changes, and has an additive effect across a broad range of patient phenotypes [7].

The LDL-C reductions seen in this study mirror those achieved with triple therapy (statin-ezetimibe-bempedoic acid) in the LAI-REACT trial [10]. Furthermore, they compare favorably to data from international trials such as EVOPACS, where HIS plus PCSK9i achieved similar target rates but were far less accessible [9]. Notably, nearly all the LDL-C reductions occurred within the first two weeks, again underscoring the value of early combination initiation rather than stepwise add-on therapy [10-12].

It is known that LDL-C tends to decrease transiently in the first two weeks after ACS (even in the absence of therapy, due to hemodilution, cytokine effect, etc.), with a rebound by four to six weeks [11,12]. However, the addition of combination therapy ensures the initial gains are not short-lived and consolidates and extends this reduction throughout the highest-risk period.

Combination therapy was very well tolerated, with no instances of therapy discontinuation for adverse events. Mild myalgias and asymptomatic CK elevation were observed at rates similar to historical controls for statin therapy alone, supporting the safety of this approach [13].

The study strongly advocates for guideline-based implementation of upfront combination therapy in all statin-naïve ACS patients, rather than waiting for failure of statin monotherapy. Early repeat LDL-C testing (e.g., at two weeks) allows for timely identification of therapy “non-responders,” who may benefit from further intensification or the addition of a third agent as per LAI-REACT and international guidance.

Limitations

This study’s strengths include its clinically relevant question, rigorous real-world patient selection at a large PCI-capable center, protocolized and frequent direct measurement of LDL-C, use of validated endpoints, and comprehensive safety monitoring throughout the follow-up period. However, significant limitations should be acknowledged: first, the relatively small, single-center sample may have limited our ability to detect less frequent outcomes and increases the risks of both Type I (false positive) and Type II (false negative) errors, particularly for secondary endpoints. Although post-hoc power analysis showed >80% power to detect the observed difference in LDL-C reduction, we may have been underpowered to detect smaller or subgroup-specific effects. Our findings should therefore be viewed as hypothesis-generating. Second, while demographics and baseline clinical characteristics appeared balanced between groups (Table 1), the non-randomized, retrospective nature of the control cohort introduces the possibility of residual confounding. Unmeasured variables, including socioeconomic status, medication adherence, and background cardiovascular risk factors, could have influenced the results. Selection bias may also have occurred, as the control group comprised only those with complete lipid profiles at baseline and week 4. To mitigate confounding, we matched the control group for age, gender, and significant comorbidities, applied strict inclusion/exclusion criteria, and used the same standardized protocol for lipid measurement at baseline and four-week follow-up. However, only a large, prospective, randomized controlled trial can eliminate such bias. Third, the follow-up was restricted to six weeks, meaning that any assessment of long-term adherence or sustained benefit is extrapolated rather than directly observed. Clinical outcome events such as recurrent myocardial infarction, stroke, or death were infrequent, and the study was not powered to evaluate these endpoints formally.

## Conclusions

This real-world cohort study demonstrates that initiating high-intensity statin plus ezetimibe at the time of ACS admission leads to larger and more rapid short-term reductions in LDL-C than high-intensity statin alone in statin-naïve patients. While these findings underscore the potential role of early combination therapy in attaining contemporary lipid targets, our results should be interpreted as supporting improvement in a validated surrogate endpoint (LDL-C), rather than direct reductions in clinical events. The observational nature and single-center design limit causal claims and generalizability; larger randomized trials with clinical endpoints are warranted.
